# Can a pragmatic responsive feeding scale be developed and applied globally?

**DOI:** 10.1111/mcn.13004

**Published:** 2020-04-15

**Authors:** Rafael Pérez‐Escamilla, Sofia Segura‐Pérez

**Affiliations:** ^1^ Yale School of Public Health Yale University New Haven Connecticut USA; ^2^ Nutrition Unit Hispanic Health Council Hartford Connecticut USA

**Keywords:** appetite, body weight, child growth, child nutrition, infancy and childhood, infant feeding behaviour, regulation

## Abstract

Responsive feeding (RF) has been recognized as necessary to prevent all forms of malnutrition including stunting and childhood obesity. Specific RF guidelines have been developed, but it is unclear how RF behaviours can be monitored systematically. Therefore, developing valid and reliable abbreviated and pragmatic RF scales is an important global priority. This is challenging, as RF is a construct with multiple dimensions including recognizing and responding to hunger and satiety cues, providing a nurturing environment during feeding episodes, and understanding how feeding needs evolve as a function of the developmental stage of the young child. Further, RF is embedded within the responsive parenting framework that in addition to RF includes sleep, soothing and play routines and the interconnections between them. A recent pioneer study conducted in a rural area of Cambodia validated an 8‐item RF scale through direct feeding observations of 6‐ to 23‐month‐old infants at home, as part of two cross‐sectional surveys conducted before and after a complementary feeding intervention. It is important for similar research to be conducted elsewhere to find out if it is possible or not to develop a core RF scale that is valid and reliable and that has adequate specificity and sensitivity for application in community studies and population surveys globally. As highlighted in this article, different definitions of RF have been used in the field; thus, it is important to reach consensus on a single definition to help move this research area forward.

1

Responsive feeding (RF) is defined in this commentary as ‘feeding practices that encourage the child to eat autonomously and in response to physiological and developmental needs, which may encourage self‐regulation in eating and support cognitive, emotional and social development’ (Pérez‐Escamilla, Segura‐Pérez, & Hall Moran, 2019). RF is increasingly being recognized as key to understanding how to help infants and young children develop lifelong healthy dietary habits and to self‐regulate food intake (Pérez‐Escamilla, Segura‐Pérez, & Lott, 2017). In addition, RF has been recognized as necessary to prevent all forms of malnutrition, including stunting, wasting and childhood obesity (Engle & Pelto, 2011; Pérez‐Escamilla & Segura‐Pérez, 2019; UNICEF, 2020). Therefore, developing valid and reliable RF measures is an important global priority (Bentley, Wasser, & Creed‐Kanashiro, 2011; Hurley, Cross, & Hughes, 2011).

The development of RF scales is challenging, as it is a construct with multiple dimensions, including recognizing and responding to hunger and satiety cues, providing a nurturing environment during feeding episodes and understanding how feeding needs evolve as a function of the developmental stage of the young child (Pérez‐Escamilla et al., 2017). Furthermore, RF falls under the umbrella of the responsive parenting framework that acknowledges that soothing, sleep and play routines are intimately intertwined with feeding routines (Black & Aboud, 2011; Pérez‐Escamilla et al., 2017). Each of these dimensions of responsive parenting require valid and reliable scales to measure them (Paul et al., 2014).

Heller and Mobley (2019) published a systematic review that included 33 instruments that assess parental RF in children up to 5 years of age. Studies were categorized into four RF domains: Food Rewards; Pressure to Eat Parental Control of Intake; Emotional Feeding; and Responsiveness to Cues/Child Autonomy. Of the 15 instruments intended for birth to 2‐year‐olds and the 28 intended for 3‐ to 5‐year‐olds, only three instruments showed rigorous validation and reliability testing (Feeding Practices and Structure Questionnaire, Comprehensive Feeding Practices Questionnaire, and Family Food Behavior Survey). The most commonly reported psychometric testing was construct validity and internal reliability. The vast majority of studies were conducted in high‐income countries, mainly in the United States and Western Europe. There were limited instruments intended for low‐income families, diverse racial and ethnic groups (Hispanic and non‐Hispanic black), fathers or other caregivers. The most frequently assessed feeding practices included Pressure to Eat, Parental Control and Food Rewards, but none of the instruments assessed all aspects of RF. Furthermore, very few of the scales were tested specifically with children under 2 years of age. The authors concluded that there is a need for more comprehensive instruments that measure all aspects of RF, for further testing in diverse populations, and further validity and reliability testing.

In their pioneer study, Sall et al. (2020) addressed several of the shortcomings identified in the review by Heller and Mobley by developing and validating an abbreviated and pragmatic RF scale that has potential to be used in community studies and population surveys. The scale was developed using data routinely collected among 6‐ to 23‐month‐old children in rural Cambodia through the ‘Opportunistic Observation Form’ from the Process for the Promotion of Child Feeding (ProPAN) package (PAHO, WHO, & UNICEF, 2013). This form is designed to identify the context of feeding behaviours among children under 2 years of age by directly observing the interaction between the caregiver and child during the child's mealtime paying special attention to RF behaviours. RF data were collected through direct observations of two children's meal episodes at home as part of two cross‐sectional surveys, conducted before and again after the implementation of a complementary feeding intervention. A meal was defined as a dish that included rice or porridge combined with any other foods such as vegetables and fish.

The baseline and endline cross‐sectional surveys were used to define an initial scale composed of four constructs and 15 indicators broken down into four themes: RF; active feeding; self‐feeding; and food situations (*N* = 243 caregiver–child pairs at baseline and 248 at endline). The behaviours were rated as positive, negative or neutral according to the age of the child; 6–11 versus 12–23 months. The 8‐item abbreviated scale was developed by removing the items without a proper fit of the initial scale and retesting its psychometric properties with confirmatory factor analyses (CFAs) and other psychometric statistics. It included two latent constructs, five indicators for RF (child served first; eats with a caregiver and family; eats in own plate; fed with utensils; and how the caregiver spent time while feeding the child, distractions) and three indicators for active feeding (the caregiver talks to the child, verbally encourage him to eat; encourages the child when he is eating well; and motivates the child to eat more using gestures/games or by demonstrating to him how to eat). The fit and reliability of the abbreviated RF scale was confirmed with both baseline and endline data.

The lessons learned from this innovative study include first the importance of using sound RF frameworks and direct observations at different time points to develop RF scales from the ground up and properly test their psychometric validity and reliability. Second, this study highlights the need to recognize that the external validity of this scale cannot be assumed. For example, the items that did not fit well the initial scale in this setting, for example, response to child refusal to eat and child self‐feeding, may be important to include in other settings (Jansen, Mallan, Nicholson, & Daniels, 2014). Because it is unreasonable to expect that a RF scale will be developed for each context, it is important for this research to continue in other settings to find out if, as it happened with the measurement of household food insecurity (Food and Agriculture Organization (FAO), undated), if it is possible to have a core pragmatic RF scale that is valid and reliable and that has adequate specificity and sensitivity globally. If successful, then, this effort would, for the first time, provide a standard way to measure an often neglected aspect of infant and young child feeding, that is *the way children are fed*, which is now recognized to affect their nutrition, health, development and wellbeing of children in the short, medium and long term. One approach could be to establish a multicountry research consortium to assess the fit of the 15 initial items included in the Cambodia study using a standard validation protocol.

Moving forward, and given the complexity of attempting to measure all possible aspects of RF through a pragmatic scale suitable for field and populations studies, it is key that researchers ask themselves first which aspects of RF they want to focus on and why, and if they also need to incorporate abbreviated scales to measure other aspects of responsive parenting directly linked with infant and young child feeding, including sleep, soothing and physical activity (Pérez‐Escamilla et al., 2017). Lastly, because as highlighted by Sall et al. (2020), different definitions of RF have been used, hence it is important to reach consensus on a single definition of this complex construct to help move this research area forward.

## CONTRIBUTIONS

All authors have read and approved the final manuscript. RP‐E wrote the first draft, and SS‐P reviewed and provided feedback that led to substantive changes to original draft.
